# Dissociação entre Biomarcadores e Imagem na Amiloidose Cardíaca do Tipo Selvagem Após Tratamento com Patisiran

**DOI:** 10.36660/abc.20240757

**Published:** 2026-04-14

**Authors:** Claudio Tinoco Mesquita, Davi Shunji Yahiro, Roberta Pereira dos Santos Coelho, Giovane Leal de Azevedo, Beatriz Robert Moreira, Régina Limongi de Figueiredo, Eliza Gripp, Diane Xavier Ávila

**Affiliations:** 1 Universidade Federal Fluminense Niterói RJ Brasil Universidade Federal Fluminense, Niterói, RJ – Brasil; 2 Hospital Pró-Cardíaco Rio de Janeiro RJ Brasil Hospital Pró-Cardíaco, Rio de Janeiro, RJ – Brasil

**Keywords:** Amiloidose, Biomarcadores, Ecocardiografia

## Introdução

A amiloidose é uma doença progressiva e complexa causada pela deposição extracelular de proteínas fibrilares insolúveis em órgãos e tecidos.^1^ Entre os diferentes tipos de amiloidose, a amiloidose cardíaca apresenta desafios únicos devido ao seu impacto direto na função, estrutura e condução elétrica do coração.^1^ Essa condição pode levar à cardiomiopatia restritiva, resultando em insuficiência cardíaca, arritmias e outras complicações potencialmente fatais.^2^ A amiloidose por transtirretina (ATTR) é uma das formas mais comuns de amiloidose cardíaca, ocorrendo tanto na forma hereditária quanto na não hereditária.^2^ A forma hereditária é causada por mutações genéticas, enquanto a forma não hereditária, conhecida como amiloidose por transtirretina do tipo selvagem (ATTRwt), afeta predominantemente adultos mais velhos e não envolve mutações genéticas.^2^

Avanços terapêuticos recentes no tratamento da amiloidose, particularmente da ATTRwt cardíaca, abriram caminhos promissores para a redução da carga amiloide em pacientes afetados. O patisiran, uma terapia baseada em interferência de RNA (RNAi), tem como alvo a síntese hepática de transtirretina, reduzindo assim os níveis circulantes da proteína transtirretina disponíveis para formar depósitos amiloides nos tecidos.^3^ Esse mecanismo representa um grande avanço, porque reduz diretamente a fonte da patologia, em vez de apenas controlar os sintomas.^4^

O monitoramento preciso da amiloidose cardíaca continua sendo um desafio, pois a progressão da doença e a resposta ao tratamento podem variar significativamente entre os pacientes. Técnicas de imagem não invasivas, como ecocardiografia e ressonância magnética cardiovascular, ganharam reconhecimento por seu potencial para avaliar a estrutura e a função cardíaca na amiloidose.^5 , 6^ No entanto, essas ferramentas ainda apresentam limitações no rastreamento confiável das alterações na carga amiloide, especialmente ao avaliar a eficácia de novas terapias. A imagem SPECT seriada com ^99m^Tc-PYP/DPD/HMDP ainda não é recomendada para esse fim.^5^ O manejo da ATTRwt cardíaca é complexo, e uma abordagem multimodal pode melhorar a avaliação da doença. Relatamos o caso de um paciente idoso com ATTRwt que apresentou redução progressiva nos parâmetros da cintilografia com pirofosfato (PYP) após tratamento com o agente de RNAi patisiran.

## Relato de caso

Um homem de 86 anos, com histórico de cirurgia de revascularização do miocárdio há 28 anos, duas angioplastias coronárias há 3 anos, hipotireoidismo controlado, síndrome da apneia-hipopneia do sono, diabetes e doença renal crônica, apresentou-se no pronto-socorro com palpitações e dispneia. Ele havia sido diagnosticado anteriormente com flutter atrial instável. Foi realizada cardioversão elétrica, após a qual ele desenvolveu graus variáveis de bloqueio atrioventricular. Os exames laboratoriais descartaram amiloidose por cadeia leve de imunoglobulina (AL), e a ATTRwt foi confirmada por cintilografia miocárdica com PYP. O teste genético para mutações no gene da transtirretina foi negativo.

Os resultados laboratoriais mostraram hematócrito de 44%, hemoglobina de 15 g/dL, contagem de leucócitos de 7.770/μL, contagem de plaquetas de 256.000/μL, hemoglobina glicada de 6,8%, glicose de 120 mg/dL, sódio de 141 mmol/L, potássio de 4,5 mmol/L, ureia de 58 mg/dL, creatinina de 1,6 mg/dL, hormônio tireoestimulante de 3,46 mUI/L, T4 livre de 1,21 ng/dL, proteína C-reativa de 0,08 mg/dL, troponina de 42,3 pg/mL e NT-proBNP de 1800 pg/mL. O monitoramento Holter em abril de 2021 revelou flutter atrial com bloqueio atrioventricular de alto grau e pausas de até 3 segundos. Um marcapasso permanente foi implantado após ablação de flutter atrial em junho de 2021. O ecocardiograma revelou volumes atriais esquerdo (94 mL) e direito (78 mL) aumentados, disfunção diastólica do ventrículo esquerdo com uma relação E/E′ de 15,2, disfunção sistólica global moderada do ventrículo esquerdo com uma deformação de −11,2%, fração de ejeção do ventrículo esquerdo de 41,5% e pressão sistólica da artéria pulmonar de 46 mmHg.

Após 2 anos de tratamento com patisiran, a insuficiência cardíaca do paciente permaneceu estável, sem alterações significativas nos parâmetros ecocardiográficos ou biomarcadores. A SPECT com ^99m^Tc-PYP foi realizada com aquisições planares 1 hora e 3 horas após a injeção. Regiões de interesse foram desenhadas manualmente sobre o coração e o tórax contralateral, e foi calculada a relação coração/contralateral (H/CL). A captação cardíaca de PYP diminuiu progressivamente com a exposição ao patisiran. Após 5 meses, a relação H/CL diminuiu de 1,62 para 1,42 e caiu ainda mais para 1,25 após 24 meses.

## Discussão

No caso relatado, a cintilografia com PYP demonstrou uma redução acentuada na captação miocárdica ao longo de 2 anos de tratamento com patisiran. Especificamente, a relação H/CL do PYP diminuiu de 1,62 na linha de base para 1,47 aos 12 meses e 1,25 aos 24 meses, sugerindo uma redução na carga amiloide miocárdica. Esses achados são consistentes com estudos recentes que indicam que terapias de RNAi, como o patisiran, podem reduzir os depósitos de amiloide miocárdico em pacientes com ATTR. Rettl et al. relataram que a terapia com RNAi reduziu significativamente a captação miocárdica quantitativa de ^99m^Tc-DPD em pacientes com cardiomiopatia hereditária por ATTR, com resultados comparáveis ao tratamento com tafamidis na cardiomiopatia por ATTRwt, sem impacto na função cardíaca, sugerindo que pode servir como uma ferramenta potencial para monitorar a resposta ao tratamento.^7^ Um estudo recente de Hung et al. mostrou uma diminuição significativa na relação volumétrica coração-pulmão em pacientes com ATTR hereditária após terapia com RNAi.^8^ Dados da subpopulação brasileira do ensaio APOLLO-B mostraram que apenas os pacientes em uso ativo de patisiran, em comparação com o placebo, demonstraram redução na captação miocárdica de ^99m^Tc-PYP, indicando benefício do direcionamento da deposição de amiloide cardíaco por meio do *knockdown* de TTR ou mecanismos imunomediados.^9^

Papathanasiou et al. estudaram 14 pacientes tratados com tafamidis por uma média de 44 meses. Cinco pacientes demonstraram regressão do grau de Perugini no ^99m^Tc-DPD, embora não tenham ocorrido alterações significativas no NT-proBNP ou nas medidas ecocardiográficas.^10^ Além disso, em uma coorte prospectiva de 23 pacientes com amiloidose cardíaca ATTR em terapia de estabilização da transtirretina, o SPECT/CT com ^99m^Tc-PYP mostrou reduções significativas nas métricas de captação miocárdica, enquanto os parâmetros ecocardiográficos e os biomarcadores permaneceram estáveis, sugerindo estabilização da doença em vez de regressão.^11^ No entanto, outros estudos recentes relataram o uso de medidas quantitativas do valor de captação padronizado (SUV) após terapia com tafamidis com benefícios significativos para a função ventricular esquerda e direita e biomarcadores cardíacos, embora sejam necessárias mais pesquisas para validar as métricas ideais e a reprodutibilidade.^12 - 14^

Embora a imagem nuclear tenha indicado uma resposta favorável ao tratamento, o mecanismo subjacente à redução da captação de PYP permanece obscuro. Uma hipótese é que as terapias de RNAi suprimem a produção de transtirretina, interrompendo assim a deposição adicional de amiloide e reduzindo indiretamente a captação do traçador. É importante considerar se a cintilografia com PYP deve ser complementada por outras modalidades para avaliar a melhora funcional e o benefício clínico geral. Além disso, vale ressaltar que o método semiquantitativo da relação H/CL apresenta limitações, visto que a confirmação da captação miocárdica é crucial para o diagnóstico e a validação prospectiva com SPECT/CT quantitativo é necessária.^15^ Para os médicos que tratam a amiloidose cardíaca, o objetivo terapêutico vai além da redução dos depósitos de amiloide, buscando melhorar a função cardíaca, aliviar os sintomas e aumentar a qualidade de vida. Rettl et al. demonstraram que a captação cardíaca quantitativa de DPD se correlaciona com a carga de amiloide miocárdica, alterações longitudinais na função cardíaca, biomarcadores cardíacos, capacidade de exercício e estágio da doença, fornecendo uma ferramenta valiosa para quantificar e monitorar a carga da doença cardíaca.^16^ Os autores sugerem que o índice de retenção de SUV pode ter valor prognóstico na cardiomiopatia por ATTRwt e que a quantificação pode servir como uma ferramenta valiosa para monitorar a carga da doença cardíaca nessa população.

No caso relatado, a diminuição da carga amiloide na cintilografia, sem melhorias correspondentes nos biomarcadores ou na função ecocardiográfica, sugere que o tratamento pode interromper a progressão da doença, mas é provável que o amiloide previamente depositado permaneça no miocárdio. Estudos adicionais de longo prazo são necessários para investigar se os traçadores com alta afinidade por amiloide são mais sensíveis do que as modalidades de imagem estabelecidas para monitorar a progressão e os desfechos da doença.

Em conclusão, uma abordagem multimodal que combine imagem, biomarcadores e avaliações funcionais pode ser considerada. Estudos longitudinais que examinem a relação entre a redução da carga amiloide na avaliação por imagem e as alterações nos desfechos clínicos, particularmente em pacientes com ATTRwt, podem fornecer informações valiosas sobre o manejo e a interpretação das respostas terapêuticas.

**Figura 1:**
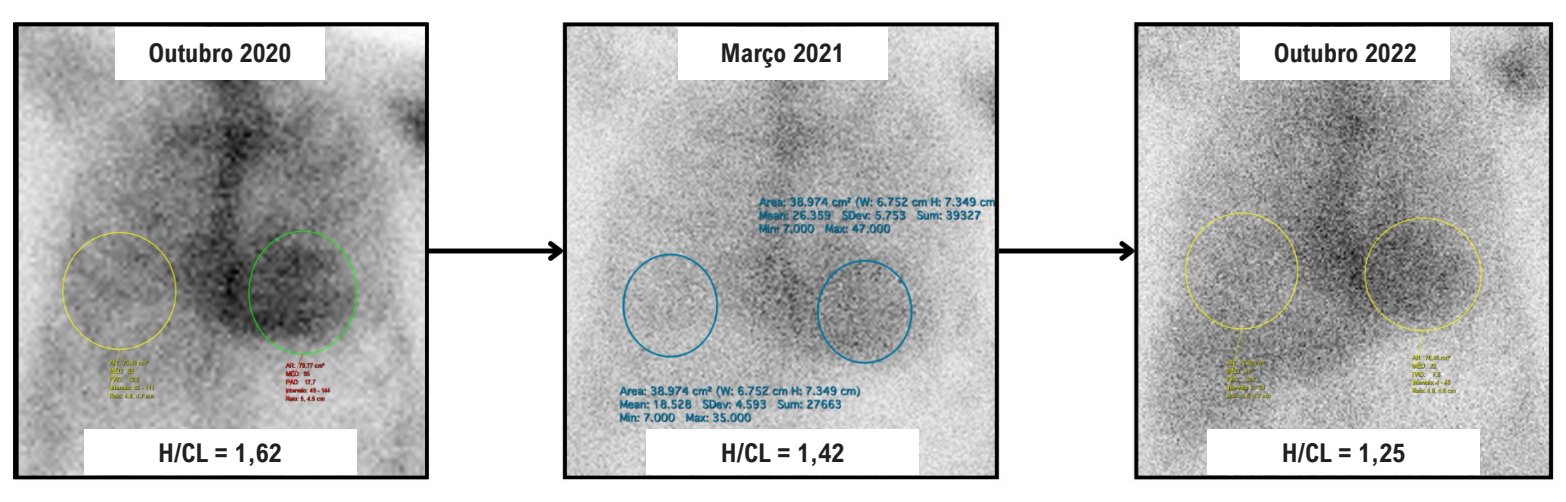
– Diminuição progressiva da relação H/CL ao longo do tratamento com patisiran. Todas as imagens mostradas foram adquiridas 3 horas após a injeção. H/CL: relação coração/contralateral.
