# Supplementary material for: Dissociação entre Biomarcadores e Imagem na Amiloidose Cardíaca do Tipo Selvagem Após Tratamento com Patisiran
Source: Arq Bras Cardiol. 2026 Apr 14;123(3):e20240757. [Article in Portuguese] doi: 10.36660/abc.20240757 (PMC13361804; doi:10.36660/abc.20240757)
Supplement: Translation

# Translation: Biomarker–Imaging Dissociation in Wild-Type Cardiac Amyloidosis after Treatment with Patisiran

**DOI:** 10.36660/abc.20240757i

**Authors:** Claudio Tinoco Mesquita, Davi Shunji Yahiro, Roberta Pereira dos Santos Coelho, Giovane Leal de Azevedo, Beatriz Robert Moreira, Régina Limongi de Figueiredo, Eliza Gripp, Diane Xavier Ávila

**Affiliations:** 1 Brazil Universidade Federal Fluminense, Niterói, RJ – Brazil; 2 Brazil Hospital Pró-Cardíaco, Rio de Janeiro, RJ – Brazil

**Keywords:** Amyloidosis, Biomarkers, Echocardiography

## Introduction

Amyloidosis is a progressive and complex disease caused by the extracellular deposition of insoluble fibrillar proteins in organs and tissues.^1^Among the different types of amyloidosis, cardiac amyloidosis poses unique challenges due to its direct impact on cardiac function, structure, and electrical conduction.^1^This condition can lead to restrictive cardiomyopathy, resulting in heart failure, arrhythmias, and other potentially life-threatening complications.^2^Transthyretin amyloidosis (ATTR) is one of the more common forms of cardiac amyloidosis, occurring in both hereditary and non-hereditary forms.^2^The hereditary form is caused by genetic mutations, whereas the non-hereditary form, known as wild-type transthyretin amyloidosis (ATTRwt), predominantly affects older adults and does not involve genetic mutations.^2^

Recent therapeutic advances in the treatment of amyloidosis, particularly cardiac ATTRwt, have opened promising avenues for reducing amyloid burden in affected patients. Patisiran, an RNA interference (RNAi)-based therapy, targets the hepatic synthesis of transthyretin, thereby lowering circulating transthyretin protein levels available to form amyloid deposits in tissues.^3^This mechanism represents a major advance because it directly reduces the source of the pathology rather than merely managing symptoms.^4^

Accurate monitoring of cardiac amyloidosis remains challenging, as disease progression and treatment response can vary significantly among patients. Noninvasive imaging techniques such as echocardiography and cardiovascular magnetic resonance have gained recognition for their potential to evaluate cardiac structure and function in amyloidosis.^5 , 6^However, these tools still have limitations in reliably tracking changes in amyloid burden, especially when assessing the effectiveness of novel therapies. Serial SPECT imaging with^99m^Tc-PYP/DPD/HMDP is not yet recommended for this purpose.^5^Management of cardiac ATTRwt is complex, and a multimodal approach may improve disease evaluation. We report the case of an elderly man with ATTRwt who demonstrated a progressive reduction in pyrophosphate (PYP) scintigraphy parameters after treatment with the RNAi agent patisiran.

## Case report

An 86-year-old man with a history of coronary artery bypass surgery 28 years prior, two coronary angioplasties 3 years earlier, controlled hypothyroidism, sleep apnea–hypopnea syndrome, diabetes, and chronic kidney disease presented to the emergency department with palpitations and dyspnea. He had previously been diagnosed with unstable atrial flutter. Electrical cardioversion was performed, after which he developed varying degrees of atrioventricular block. Laboratory testing ruled out immunoglobulin light-chain (AL) amyloidosis, and ATTRwt was confirmed by myocardial PYP scintigraphy. Genetic testing for transthyretin mutations was negative.

Laboratory results showed hematocrit 44%, hemoglobin 15 g/dL, leukocyte count 7,770/μL, platelet count 256,000/μL, glycated hemoglobin 6.8%, glucose 120 mg/dL, sodium 141 mmol/L, potassium 4.5 mmol/L, urea 58 mg/dL, creatinine 1.6 mg/dL, thyroid-stimulating hormone 3.46 mIU/L, free T4 1.21 ng/dL, C-reactive protein 0.08 mg/dL, troponin 42.3 pg/mL, and NT-proBNP 1800 pg/mL. Holter monitoring in April 2021 revealed atrial flutter with high-degree atrioventricular block and pauses of up to 3 seconds. A permanent pacemaker was implanted after atrial flutter ablation in June 2021. Echocardiography revealed enlarged left (94 mL) and right atrial volumes (78 mL), left ventricular diastolic dysfunction with an E/E′ ratio of 15.2, moderate global left ventricular systolic dysfunction with a strain of −11.2%, left ventricular ejection fraction of 41.5%, and pulmonary artery systolic pressure of 46 mmHg.

After 2 years of patisiran treatment, the patient’s heart failure remained stable, with no significant changes in echocardiographic parameters or biomarkers.^99m^Tc-PYP SPECT was performed using both 1-hour and 3-hour post-injection planar acquisitions. Regions of interest were manually drawn over the heart and contralateral chest, and the heart-to-contralateral (H/CL) ratio was calculated. Cardiac PYP uptake decreased progressively with patisiran exposure. After 5 months, the H/CL ratio decreased from 1.62 to 1.42 and further declined to 1.25 after 24 months.

## Discussion

In this case, PYP scintigraphy demonstrated a marked reduction in myocardial uptake over 2 years of patisiran treatment. Specifically, the H/CL PYP ratio decreased from 1.62 at baseline to 1.42 at 12 months and 1.25 at 24 months, suggesting a reduction in myocardial amyloid burden. These findings are consistent with recent studies indicating that RNAi therapies such as patisiran can reduce myocardial amyloid deposits in patients with ATTR. Rettl et al. reported that RNAi therapeutics significantly reduced^99m^Tc-DPD quantitative myocardial uptake in patients with hereditary ATTR cardiomyopathy, with results comparable to tafamidis treatment in ATTRwt cardiomyopathy, without impact on cardiac function, suggesting that it may serve as a potential tool to monitor treatment response.^7^A recent study by Hung et al. showed a significant decrease in volumetric heart-to-lung ratio in patients with hereditary ATTR after RNAi therapy.^8^Data from the Brazilian subpopulation of the APOLLO-B trial showed that only patients on active patisiran, compared with placebo, demonstrated reduced myocardial^99m^Tc-PYP uptake, indicating a benefit of targeting cardiac amyloid deposition by TTR knockdown or immune-mediated mechanisms.^9^

Papathanasiou et al. studied 14 patients treated with tafamidis for an average of 44 months. Five patients demonstrated regression of Perugini grade on^99m^Tc-DPD, although there were no significant changes in NT-proBNP or echocardiographic measures.^10^Moreover, in a prospective cohort of 23 patients with ATTR cardiac amyloidosis on transthyretin stabilization therapy,^99m^Tc-PYP SPECT/CT showed significant reductions in myocardial uptake metrics, while echocardiographic parameters and biomarkers remained stable, suggesting disease stabilization rather than regression.^11^However, other recent studies have reported the use of quantitative standardized uptake value (SUV) measurements after tafamidis therapy with significant benefits for left and right ventricular function and cardiac biomarkers, though further research is needed to validate optimal metrics and reproducibility.^12^

Although nuclear imaging indicated a favorable response to treatment, the mechanism underlying reduced PYP uptake remains unclear. One hypothesis is that RNAi therapies suppress transthyretin production, thereby interrupting further amyloid deposition and indirectly reducing tracer uptake. It is important to consider whether PYP scintigraphy should be complemented by other modalities to assess functional improvement and overall clinical benefit. Additionally, it is worth noting that the semiquantitative H/CL ratio method has limitations, as myocardial uptake confirmation is crucial for diagnosis, and prospective validation using quantitative SPECT/CT is necessary.^15^For clinicians managing cardiac amyloidosis, the therapeutic goal extends beyond reducing amyloid deposits to improving cardiac function, alleviating symptoms, and enhancing quality of life. Rettl et al. demonstrated that quantitative cardiac DPD uptake correlates with myocardial amyloid load, longitudinal cardiac function, cardiac biomarkers, exercise capacity, and disease stage, providing a valuable tool to quantify and monitor cardiac disease burden.^16^The authors suggest that the SUV retention index may have prognostic value in ATTRwt cardiomyopathy and that quantification may serve as a valuable tool for monitoring cardiac disease burden in this population.

In our case, decreased amyloid load on scintigraphy without corresponding improvements in biomarkers or echocardiographic function suggests that treatment may interrupt disease progression, but previously deposited amyloid likely remains within the myocardium. Further long-term studies are necessary to investigate whether amyloid-avid tracers are more sensitive than established imaging modalities for monitoring disease progression and outcomes.

In conclusion, a multimodal approach combining imaging, biomarkers, and functional assessments may be considered. Longitudinal studies examining the relationship between reduced amyloid burden on imaging and changes in clinical outcomes, particularly in patients with ATTRwt, could provide valuable insights into the management and interpretation of therapeutic responses.

**Figure 1:** – Progressive decrease in H/CL ratio over the course of patisiran treatment. All images shown were acquired at 3 hours post-injection. H/CL: heart-to-contralateral.
